# Instruments for Assessing Risk of Bias and Other Methodological Criteria of Animal Studies: Omission of Well-Established Methods

**DOI:** 10.1289/ehp.1307727

**Published:** 2014-03-01

**Authors:** Nancy B. Beck, Richard A. Becker, Alan Boobis, Dean Fergusson, John R. Fowle, Julie Goodman, Sebastian Hoffmann, Manoj Lalu, Marcel Leist, Martin L. Stephens

**Affiliations:** 1 Regulatory and Technical Affairs, American Chemistry Council, Washington, DC, USA; 2 Department of Medicine, Imperial College, London, United Kingdom; 3 Ottawa Hospital Research Institute, University of Ottawa, Ontario, Canada; 4 Science to Inform, LLC, Pittsboro, North Carolina, USA; 5 Gradient, Cambridge, Massachusetts, USA; 6 seh consulting + services, Paderborn, Germany; 7 The Ottawa Hospital, University of Ottawa, Ontario, Canada; 8 University of Konstanz, Germany; 9 Center for Alternatives to Animal Testing, John Hopkins Bloomberg School of Public Health, Baltimore, Maryland, USA

In response to the systematic review by Krauth et al. (2013) of instruments for assessing animal toxicology studies for risk of bias and other aspects of quality, we propose the need for a broader perspective when appraising—and hopefully improving—such studies.

Krauth et al. (2013) reviewed 30 instruments, 4 of which were designed for environmental toxicology studies used to evaluate human and ecological health hazards. The authors noted that these instruments were derived from preclinical pharmaceutical research in animal models. Many of these instruments focus on efficacy and not toxicity, and—as acknowledged by the authors—they may have limited potential application in environmental health research because they often have criteria that are not relevant to hazard and risk assessments.

Based on these 30 instruments, Krauth et al. concluded that a limited number of risk of bias assessment criteria have been empirically tested for animal research, including randomization, concealment of allocation, blinding, and accounting for all animals. However, the authors did not discuss which elements of risk of bias criteria have been empirically tested, nor did they discuss how they were tested, leaving the reader with no information on their reliability or usefulness.

We would like to bring the readers’ attention to several other important publications in environmental chemical health hazard assessment that are pertinent to this topic (Ågerstrand et al. 2011; Hulzebos et al. 2010; Schneider et al. 2009), along with a U.S. Environmental Protection Agency (EPA) approach developed under the High Production Volume Challenge (U.S. EPA 1999b) as well as relevant and potentially eligible guidance developed by the U.S. EPA (1999a) and the Food and Drug Administration (FDA 2003). In addition, the majority of the procedures specified in Good Laboratory Practices and regulatory *in vivo* toxicity test guidelines (e.g., U.S. EPA 2013; Organisation for Economic Co-operation and Development 1999) were specifically developed to minimize systematic errors, assure high quality data and produce scientifically reliable studies.

These additional publications describe design, conduct, and reporting criteria that form the basis of the methodologies employed globally to assure quality and reliability of *in vivo* toxicological investigations for regulatory assessment of human and ecological health hazards. Because the application of systematic review and related evidence-based approaches in toxicology is still in its infancy, it is especially important at this time to recognize the contributions of these publications.

The omission of these publications by Krauth et al. could have major science policy implications. The National Toxicology Program (NTP) (whose parent organization, the National Institute of Environmental Health Sciences, funded the research of Krauth et al.) has begun relying on Krauth et al. (2013) to identify elements of risk of bias in evaluating animal studies of environmental agents as part of its systematic reviews for assessing health effects (NTP 2013a, 2013b). The reliance on criteria that have not been transparently empirically tested instead of well-established methodological criteria developed by authoritative national and international organizations could result in biased systematic reviews that ultimately lead to regulations or classifications not supported by the science.

We suggest that further work is warranted in pulling together published perspectives on how to evaluate study quality in animal toxicology studies. Issues in appraising such studies for evaluating environmental hazards to humans and wildlife go well beyond those of human clinical trials, and would benefit from collaboration of experts in animal toxicology with experts in human clinical trials of medical interventions and human epidemiology.
