# Erratum: “Instruments for Assessing Risk of Bias and Other Methodological Criteria of Animal Studies: Omission of Well-Established Methods”

**DOI:** 10.1289/ehp.122-A234

**Published:** 2014-09-01

In the letter “Instruments for Assessing Risk of Bias and Other Methodological Criteria of Animal Studies: Omission of Well-Established Methods” by Beck et al. [Environ Health Perspect 122:A66–A67 (2014); http://dx.doi.org/10.1289/ehp.1307727], information for Alan Boobis and Marcel Leist was omitted from the Competing Financial Interests declaration. The complete declaration is as follows:

None of the authors received specific financial support or honorarium as compensation for developing this letter. Several authors are members of the Evidence-Based Toxicology Collaboration (EBTC), and M.L. Stephens and S. Hoffmann serve as the secretariats for the North American and European EBTC Steering Committees, respectively, for which they are compensated for their time. The EBTC’s overall aims are to improve toxicological decision making, facilitate the modernization of the toxicological toolbox, and reinvigorate the safety sciences (see http://www.ebtox.com). S. Hoffmann, J.R. Fowle III, and J. Goodman are consultants and have worked on a range of toxicity and risk assessment issues for a wide variety of clients. R.A. Becker and N.B. Beck are employed by the American Chemistry Council, a trade association of chemical manufacturers. A. Boobis, D. Fergusson, M. Lalu, and M. Leist are employed by institutes of higher education. In the past 3 years, A. Boobis and M. Leist have worked on a range of toxicity and risk assessment issues for a number of clients; this has included some consultancies.

The authors regret the error.

